# The interactive effect of fungicide residues and yeast assimilable nitrogen on fermentation kinetics and hydrogen sulfide production during cider fermentation

**DOI:** 10.1002/jsfa.8096

**Published:** 2016-11-17

**Authors:** Thomas F Boudreau, Gregory M Peck, Sean F O'Keefe, Amanda C Stewart

**Affiliations:** ^1^ Department of Food Science and TechnologyVirginia Polytechnic Institute and State UniversityHABB1 Room 401, 1230 Washington Street SWBlacksburgVA24060USA; ^2^ School of Integrative Plant ScienceHorticulture SectionCornell University, 121 Plant Science BuildingIthacaNY14853USA

**Keywords:** cider fermentation, apple fungicides, fermentation rate, hydrogen sulfide, fenbuconazole, fludioxonil

## Abstract

**BACKGROUND:**

Fungicide residues on fruit may adversely affect yeast during cider fermentation, leading to sluggish or stuck fermentation or the production of hydrogen sulfide (H_2_S), which is an undesirable aroma compound. This phenomenon has been studied in grape fermentation but not in apple fermentation. Low nitrogen availability, which is characteristic of apples, may further exacerbate the effects of fungicides on yeast during fermentation. The present study explored the effects of three fungicides: elemental sulfur (S^0^) (known to result in increased H_2_S in wine); fenbuconazole (used in orchards but not vineyards); and fludioxonil (used in post‐harvest storage of apples).

**RESULTS:**

Only S^0^ led to increased H_2_S production. Fenbuconazole (≥0.2 mg L^−1^) resulted in a decreased fermentation rate and increased residual sugar. An interactive effect of yeast assimilable nitrogen (YAN) concentration and fenbuconazole was observed such that increasing the YAN concentration alleviated the negative effects of fenbuconazole on fermentation kinetics.

**CONCLUSION:**

Cidermakers should be aware that residual fenbuconazole (as low as 0.2 mg L^−1^) in apple juice may lead to stuck fermentation, especially when the YAN concentration is below 250 mg L^−1^. These results indicate that fermentation problems attributed to low YAN may be caused or exacerbated by additional factors such as fungicide residues, which have a greater impact on fermentation performance under low YAN conditions. © 2016 The Authors. *Journal of The Science of Food and Agriculture* published by John Wiley & Sons Ltd on behalf of Society of Chemical Industry.

## INTRODUCTION

Fungicide applications in orchards are essential for controlling diseases and thus ensuring sufficient fruit quality for cider production. However, fungicide residues can negatively impact yeast biology and therefore fermentation performance and subsequent cider quality. In the USA, unfermented apple juice is commonly referred to as ‘cider’; however, in the present study, as in the global cider industry, the term ‘cider’ refers to the alcoholic beverage resulting from the fermentation of apple juice. The cidermaking process may be especially prone to increased fungicide residues because of the use of fungicides as post‐harvest storage protectants, as well as the fact that, unlike grapes used in winemaking, apples used in cidermaking may be grown in the same orchard with multiple market destinations. These factors, combined with wide variation in practices for washing fruit prior to juicing, could contribute to the presence of residual fungicides in the fermenting juice.

Fungicide residues may contribute to adverse fermentation conditions and the occurrence of objectionable off‐aromas, including hydrogen sulfide (H_2_S). H_2_S is a common off‐aroma occurring during alcoholic fermentation that negatively affects the quality of finished ciders. The causes of H_2_S production during fermentation are numerous and have complex interactive effects that remain a topic of current research. Factors including yeast strain^1^, ^2^ and yeast assimilable nitrogen (YAN)^3^, ^4^, ^5^ concentration, as well as the composition, temperature and deficiencies of other yeast nutrients, such as biotin and pantothenic acid,^6^ have all been shown to contribute to overall H_2_S production in wine fermentation. Certain fungicides are also known to increase H_2_S production in wine fermentation and significantly impact wine flavor,^7^ although the impact of fungicide residues has not been thoroughly examined in cider fermentation. Similarly, insufficient YAN in the fermenting juice contributes to sluggish and/or stuck fermentations^8^, ^9^, ^10^ and reduced wine quality through the production of higher alcohols and thiols combined with a decreased production of desirable esters and long‐chain fatty acids.^8^ The interactive effects of YAN concentration and fungicide residue could reasonably be expected to significantly impact cider quality in much the same way as in grape‐based wine production.

Elemental sulfur (S^0^) can be used in orchards and is commonly used in those that are organically‐managed to limit powdery mildew incidence. The mode of action as a fungicide is not fully understood, although it is known that S^0^ acts as an electron receptor in fungal respiratory chains and stimulates respiratory activities.^11^ Sulfur is also likely related to oxidation of sulphydryl groups in important mitochondrial respiratory enzymes^12^. S^0^ residues can be toxic to predominant native yeast found on grapes;^13^ however, S^0^ is not toxic to strains of *Saccharomyces cerevisiae* even at concentrations of 200 mg L^−1^, which is a level far greater than the reported residual S^0^ concentrations in grapes or apples.^14^ Although non‐toxic to yeast at practical residual concentrations, extensive research has indicated that S^0^ residue can lead to substantial increases in H_2_S production during wine fermentation.^15^, ^16^, ^17^ In grape or apple juice fermentations, S^0^ can non‐enzymatically react with reducing compounds in the fermenting juice to form H_2_S.^18^ S^0^ residues at concentrations as low as 1 mg L^−1^ have been shown to increase H_2_S production during wine fermentation.^19^ It is common for residues on fruit and in juice to greatly exceed these concentrations. One study found that residue on Cabernet Sauvignon berries was 1.5 µg g^−1^ S^0^ (approximately 1.7 mg L^−1^ S^0^ in must) at harvest but exceeded 12 µg g^−1^ S^0^ (approximately 13.5 mg L^−1^ S^0^ in must) at 1 month after application on Pinot Noir berries.^20^ The lack of data concerning the persistence of residual S^0^ in orchard systems leads to increased risk of residual S^0^ in apple juice prior to fermentation, as well as subsequent formation of H_2_S during yeast metabolism, which can negatively impact cider quality.

The impact of other fungicide residues on yeast growth and metabolism and the resulting cider quality is not well understood. Fenbuconazole is a common fungicide that is used to prevent several plant diseases, including powdery mildew, leaf blotch and various rot diseases in both apple and grape production. Fenbuconazole is a sterol inhibitor, specifically a demethylation inhibitor that prevents the biosynthesis of ergosterol in fungal plasma membranes.^21^ Currently, there are no data available on residual fenbuconazole in apple or grape juice, nor in finished wines or ciders. In a US Department of Agriculture (USDA) survey, 2.3% of apple samples were found to have detectable levels of residual fenbuconazole ranging from 0.008 to 0.015 mg L^−1^.^22^ Fludioxonil is a broad‐spectrum fungicide used frequently in the post‐harvest storage of apples, and it operates by interfering with the signal transduction pathways of fungi.^23^ It has been found to inhibit spore germination in *Botrytis cenera*.^24^ Residues of fludioxonil have been cited to be as high as 0.15 mg L^−1^ in finished wines,^25^ although residues have not been quantified in grape juice, apple juice or ciders. The USDA found that 16.9% of apple samples contained measurable fludioxonil residues, which ranged from 0.025 to 1.0 mg L^−1^.^22^ Similarly, the impact of these fungicides on cider fermentation or finished cider or wine quality has not been studied extensively. García *et al*.^26^ found that residual fludioxonil in Airen grape must at concentrations as low as 1 mg L^−1^ significantly alters the volatile fraction of wine aromas, although other flavor impacts are unknown, including the impact on the production of H_2_S by yeast during fermentation.

The present study aimed to determine the impact of fungicide residues on fermentation kinetics and the production of H_2_S during apple cider fermentation. Laboratory scale fermentations were used to compare the effect of three fungicides [elemental sulfur (S^0^), fludioxonil and fenbuconazole] on fermentation kinetics and H_2_S production by two yeast strains commonly used in cider production.

## MATERIALS AND METHODS

### Apple juice

Commercially produced pasteurized apple juice was used to ensure the consistency of juice across samples and to best represent large‐scale cidermaking practices. The juice used was White House Fresh Pressed Natural Apple Juice (National Fruit Product Co., Winchester, VA, USA). Multiple bottles of the juice were combined into one homogenous lot then stored in 1‐L aliquots at −20 °C until use, and then thawed to 22 °C prior to yeast inoculation. The juice was measured to have 12.9 °Brix by refractometer, pH 3.7 and titratable acidity 3.4 g L^−1^ malic acid equivalent using standard methods,^27^ and 53 mg L^−1^ YAN. YAN was quantified using commercially available Megazyme (Wicklow, Ireland) kits for primary amino nitrogen (K‐PANOPA) and ammonium ion concentration (Ammonia‐Rapid).

### Fungicide additions

S^0^ was obtained in the form of Microthiol Disperss fungicide (80% sulfur; Nufarm Australia Ltd, Victoria, Australia) and added at concentrations of 5 and 20 mg L^−1^ S^0^. Fludioxonil was obtained in the form of Scholar SC fungicide (20.4% fludioxonil; Syngenta Crop Protection LLC, Greensboro, NC, USA) and added at concentrations of 0.2 and 0.4 mg L^−1^ fludioxonil. Fenbuconazole was obtained in the form of Indar 2 F fungicide (23.5% fenbuconazole; Dow Agrosciences LLC, Indianapolis, IN, USA) and added at concentrations of 2.5 and 5.0 mg L^−1^ fenbuconazole. Commercially available juice with no fungicides added was used as a control. The final fungicide concentrations in juice were not confirmed through analysis of juice after fungicide addition, which is a limitation of the present study. Furthermore, the original juice (commercially available apple juice) was not screened for fungicide residue. The results of the present study should therefore be interpreted from the perspective that the reported concentrations of fungicides added represent an additional amount to any fungicide residue possibly present in the original juice. The USDA stated limits for fungicide residues in apple juice are 0.4 mg L^−1^ for fludioxonil and 5.0 mg L^−1^ for fenbuconazole, which were set as the maximum added concentrations studied in the present study. Interactive effects of YAN and fenbuconazole residues were examined by adding YAN to the apple juice at concentrations of 25 mg N L^−1^ in the form of Fermaid K (Scott Laboratories, Inc., Petaluma, CA, USA) for the control, and further supplementing base apple juice with 100 and 200 mg N L^−1^ using diammonium phosphate to 153 and 253 mg N L^−1^ for the medium and high YAN concentration treatments (Scott Laboratories, Inc.). Each treatment was then supplemented with fenbuconazole at concentrations of 0 (control), 0.2 and 0.4 mg L^−1^ each.

### Fermentations

Experimental fermentations were carried out in triplicate with two yeast strains for each treatment. Prise de Mousse *Saccharomyces bayanus* EC1118 (Lallemand, Montreal, Canada) was selected to represent a low‐H_2_S producing strain and Montrachet *S. cerevisiae* UCD522 (Lallemand, Montreal, Canada) was selected to represent a high‐H_2_S producing strain. Juice was inoculated with 0.05 g of the active dry yeast rehydrated in 35 °C water for 20 min. Yeast nutrient was added in the form of Fermaid K (Scott Laboratories, Inc.) to all fermentations at concentrations of 25 mg L^−1^. This added an additional 25 mg L^−1^ nitrogen to the juice, bringing total juice YAN to 78 mg L^−1^ for the control because the endogenous 53 mg N L^−1^ in the apple juice was not sufficient to complete fermentation. Fermentations were carried out in accordance with the method described by Ugliano and Henschke^28^ for rapid determination of H_2_S formation during alcoholic fermentation. These were conducted in 250‐mL Erlenmeyer flasks fitted with a one‐hole rubber stopper to which a H_2_S detector tube was affixed (see below). Fermentations were not aerated but were stirred twice per day at 800 r.p.m. for 5 min to prevent yeast settling. Fermentations were carried out at 18 °C in triplicate. The fermentation rate was monitored by measuring the mass of the fermentation vessel as a proxy for CO_2_ evolution. Finished cider was analyzed to determine pH, titratable acidity, residual YAN and residual sugar. Residual sugar was analyzed using a d‐fructose/d‐glucose (K‐FRUGLU) enzymatic kit (Megazyme).

### H_2_S detector tubes

The H_2_S detection and quantification method was as described by Ugliano and Henschke.^28^ Detector tubes were obtained from Komyo Kitagawa (Tokyo, Japan). Tubes were inserted into a one‐hole rubber stopper to obtain a gas‐tight seal. CO_2_ produced during fermentation carried H_2_S through the detector tube. H_2_S reacts with the lead acetate (Tube 120SB, 120SD) or silver nitrate (Tube 120SF) contained in the tube, creating a discolored band. The different tubes have different capacities for total H_2_S quantification, and were selected based on total H_2_S production in a given treatment as determined by preliminary experimentation. The length of the discolored band is proportional to the amount of purged H_2_S. Readings were taken twice daily to determine the H_2_S production rate. If, at any reading point, the tube appeared to be near saturation, the tube was replaced with a new tube. This method may have allowed a small amount of H_2_S gas to escape, although it was employed consistently across treatments in the present study.

### Determination of fermentation rate, duration and H_2_S production rate

Maximum fermentation rate was determined by taking the slope of the fermentation curve during the exponential phase of yeast growth corresponding to the highest constant rate of CO_2_ production, as reported previously.^29^ Steeper slopes correspond to faster fermentation rate. Fermentations were determined to be complete when the CO_2_ production rate decreased to less than 0.2 g day^−1^. Fermentation duration is expressed as total hours starting from inoculation. Total H_2_S production was determined by calculating the sum of H_2_S production over the time course of fermentation. To determine the relative rate of H_2_S production over the time course of fermentation, the fermentation duration was divided into four quartiles of equal time. The percentage of H_2_S produced in each quartile out of the total H_2_S produced for a given fermentation was compared to determine whether there was a significant difference in the H_2_S production over the time course of fermentation across treatments.

### Statistical analysis

Values were compared using a one‐way analysis of variance (ANOVA), with *P* < 0.05 being considered statistically significant, followed by parametric mean testing using Tukey's honestly significant difference (HSD) using Prism, version 6 (GraphPad, La Jolla, CA, USA). Analyses comparing the interaction between yeast strain/YAN concentration and fungicide residues were analyzed using a two‐way ANOVA, with *P* < 0.05 being considered statistically significant, and post‐hoc testing by Tukey's HSD.

## RESULTS AND DISCUSSION

### Fermentation kinetics

#### Elemental sulfur

Regardless of the concentration added, S^0^ did not affect the fermentation duration, rate, total CO_2_ production or residual sugar with yeast strain EC1118 (Table 1). At the concentration of 5 mg L^−1^, S^0^ had no effect on fermentation duration, fermentation rate or residual sugar with either yeast strain (Tables 1 and 2). The 5 mg L^−1^ S^0^ treatment resulted in decreased total CO_2_ production by UCD 522, although there was no effect in the fermentations with EC1118. The 20 mg L^−1^ S^0^ treatment using strain UCD 522 containing added S^0^ at 20 mg L^−1^ was longer in duration (*P* < 0.05) by 25 h on average, and also had a lower maximum fermentation rate (*P* < 0.05) and lower total CO_2_ production than the control, although this effect was not observed for yeast strain EC1118 at the same S^0^ concentration. The interaction effect of yeast strain and S^0^ was significant with regards to fermentation duration (*P* = 0.0002, data not shown). Fermentation duration is known to differ among yeast strains^30^ and the results of the present study show that effect of S^0^ on fermentation rate also differs across yeast strains. The significance of this interaction effect supports the need to employ at least two yeast strains in studies investigating the impact of juice chemistry on fermentation performance. This also emphasizes the importance of considering the yeast strain when applying research findings to cider production. The residual sugar concentration was not different for any S^0^ treatment or yeast strain. CO_2_ production is directly correlated with the fermentation rate because CO_2_ evolution occurs concomitantly with sugar consumption by yeast. The lack of a significant difference in residual sugar (RS) at the same time as significant differences in CO_2_ production being observed in the added S^0^ conditions for fermentations conducted by UCD522 is likely a result of the higher level of precision in measurements for CO_2_ production by mass compared to the RS measurement by enzymatic assay. Taken together, these results suggest that cidermakers should be aware that residual S^0^ above 20 mg L^−1^ in juice can result in a fermentation duration of up to 1 day longer at 18 °C, although this should not impact on residual sugar concentration in the finished cider. The fermentation curves showing CO_2_ production over the time course of fermentation in Fig. 2(A, B) further illustrate this conclusion. The observed impact of S^0^ on fermentation kinetics as a whole is consistent with previous research indicating that sulfur residue does not significantly impact fermentation kinetics of *S. cerevisiae* during fermentation.^14^

**Table 1:** Fermentation rate, fermentation duration, total CO_2_ production and residual sugar for fermentations using yeast strain EC1118

Experimental treatment	Fermentation rate (g CO_2_ h^−1^)	Fermentation duration (h)	Total CO_2_ production (g)	Residual sugar (g L^−1^)
Control	0.073 ± 0.002 a	259 ± 0 a	9.88 ± 0.13 a	0.05 ± 0.02 a
Sulfur, 5	0.071 ± 0.000 a	259 ± 0 a	9.63 ± 0.16 a	0.02 ± 0.01 a
Sulfur, 20	0.072 ± 0.001 a	256 ± 0 a	9.74 ± 0.23 a	0.03 ± 0.00 a
Control	0.073 ± 0.002 a	259 ± 0 a	9.88 ± 0.13 a	0.05 ± 0.02 a
Fludioxonil, 2.5	0.071 ± 0.003 a	251 ± 0 a	9.85 ± 0.26 a	0.04 ± 0.02 a
Fludioxonil, 5.0	0.068 ± 0.006 a	259 ± 0 a	10.08 ± 0.34 a	0.05 ± 0.03 a
Control	0.073 ± 0.002 a	259 ± 0 a	9.88 ± 0.13 a	0.05 ± 0.02 a
Fenbuconazole 0.2	0.063 ± 0.003 b	306 ± 24 b	9.36 ± 0.05 b	3.32 ± 2.92 a
Fenbuconazole, 0.4	0.059 ± 0.000 b	314 ± 13 b	9.12 ± 0.21 b	9.26 ± 0.98 b

Values expressed as the mean ± SD.

Values marked with different lowercase letters are significantly different from the control within a specified fungicide treatment (ANOVA with Tukey's HSD).

Fungicide concentrations are expressed as mg L^−1^.

**Table 2:** Fermentation rate, fermentation duration, total CO_2_ production and residual sugar for fermentations using yeast strain UCD522

Experimental treatment	Fermentation rate (g CO_2_ h^−1^)	Fermentation duration (h)	Total CO_2_ production (g)	Residual sugar (g L^−1^)
Control	0.071 ± 0.001 a	259 ± 0 a	9.90 ± 0.09 a	0.07 ± 0.01 a
Sulfur, 5	0.070 ± 0.001 a	258 ± 10 a	9.63 ± 0.03 b	0.40 ± 0.07 a
Sulfur, 20	0.067 ± 0.002 b	284 ± 0 b	9.60 ± 0.14 b	0.06 ± 0.00 a
Control	0.071 ± 0.001 a	259 ± 0 a	9.90 ± 0.09 a	0.07 ± 0.01 a
Fludioxonil, 2.5	0.074 ± 0.002 ab	259 ± 0 a	9.76 ± 0.15 a	0.10 ± 0.02 a
Fludioxonil, 5.0	0.075 ± 0.002 b	259 ± 0 a	9.87 ± 0.14 a	0.07 ± 0.02 a
Control	0.071 ± 0.001 a	259 ± 0 a	9.90 ± 0.09 a	0.07 ± 0.01 a
Fenbuconazole, 0.2	0.065 ± 0.001 b	318 ± 9 b	9.94 ± 0.13 a	2.40 ± 1.19 b
Fenbuconazole, 0.4	0.061 ± 0.001 c	322 ± 13 b	9.41 ± 0.06 b	7.72 ± 0.99 c

Values expressed as the mean ± SD.

Values marked with different lowercase letters are significantly different from the control within a specified fungicide treatment (ANOVA with Tukey's HSD).

Fungicide concentrations are expressed as mg L^−1^.

#### Fludioxonil

The addition of fludioxonil did not significantly impact fermentation rate or duration in either yeast strain, with one exception. The 5 mg L^−1^ addition of fludioxonil slightly but significantly increased fermentation rate in the strain UCD522 (Table 2). No difference in CO_2_ production or RS was observed for either yeast strain in fermentations containing added fludioxonil (Table 2). Fludioxonil did not impact the time course of CO_2_ production in either yeast strain (Fig. 2C, D). Taken together from a practical perspective, these results suggest that fludioxonil does not significantly impact fermentation kinetics for EC1118 or UCD522.

#### Fenbuconazole

**Figure 1:**
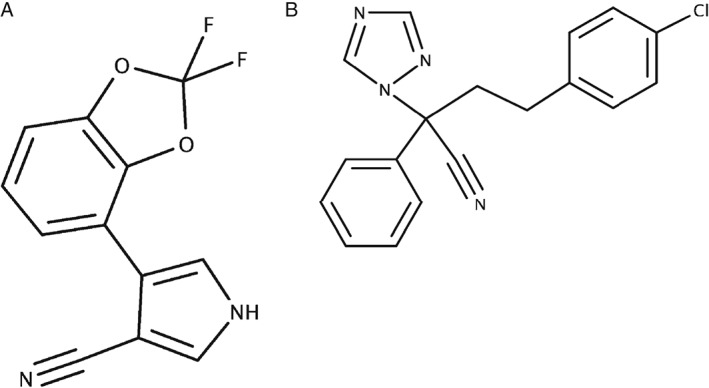
Chemical structures of (A) fludioxonil and (B) fenbuconazole.

The addition of fenbuconazole increased fermentation duration (*P* < 0.05) and decreased fermentation rate (*P* < 0.01) in both yeast strains (Figures 1E, F and Tables 1 and 2). The addition of fenbuconazole at concentrations of 0.2 mg L^−1^ decreased the fermentation rate by approximately 10% on average in both strains. The fermentation rate decreased by 15% on average when fenbuconazole was added at concentrations of 0.4 mg L^−1^. Consequently, this increased fermentation duration in both strains at all concentrations (*P* < 0.05). On average, the addition of fenbuconazole increased fermentation duration by approximately 55 h regardless of yeast strain or concentration.

**Figure 2:**
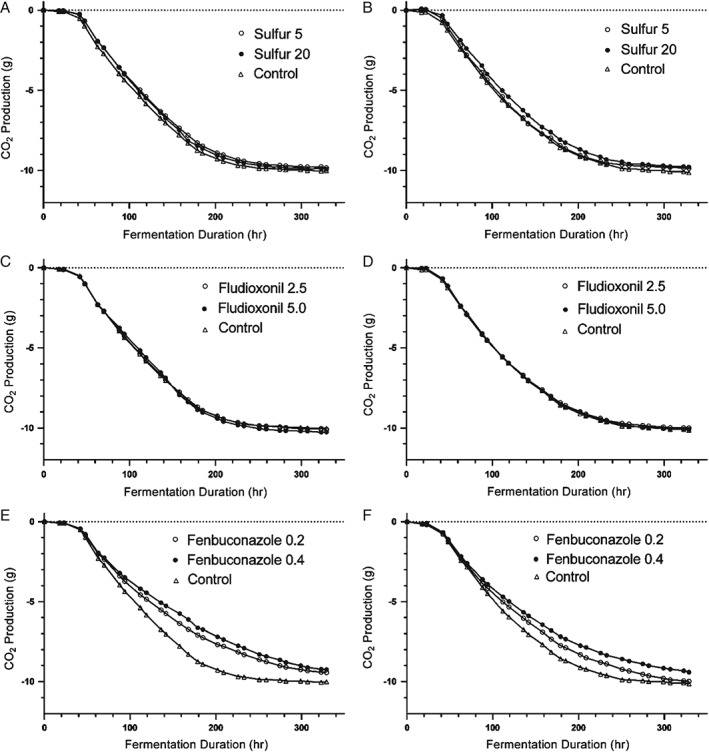
Fermentation curves for treatments containing the addition of fungicides plotted as change in the mass of the fermentation vessel as a result of CO_2_ purged from the fermenter over the time course of fermentation. The slope of the curve during the logarithmic phase of growth was used to calculate and compare maximum fermentation rates. (A), (C) and (E) Fermentations conducted by yeast strain EC1118. (B), (D) and (F) Fermentations conducted by yeast strain UCD522. Fungicide concentrations are expressed as mg L^−1^. Control fermentations contained no added fungicides.

**Figure 3:**
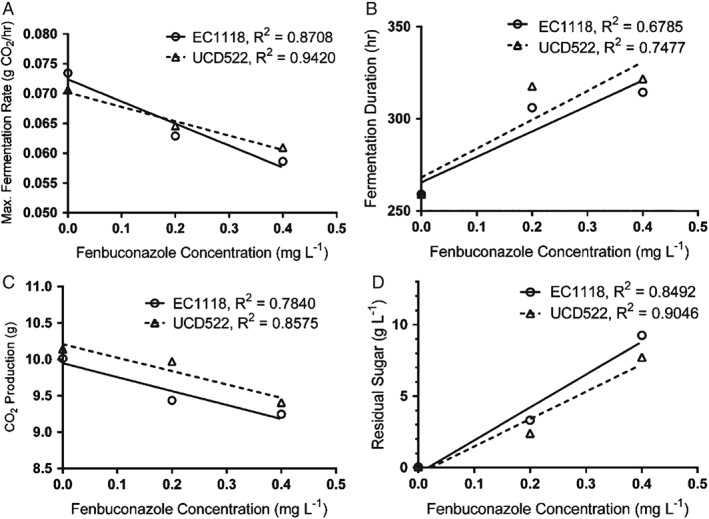
Linear regression of the maximum fermentation rate (A), fermentation duration (B), CO_2_ evolved (C) and residual sugar (D) for treatments with increasing fenbuconazole concentrations. Concentrations of fenbuconazole are expressed as mg L^−1^. All linear correlations were statistically significant (P < 0.01).

The addition of fenbuconazole decreased the amount of CO_2_ produced during fermentation for both yeast strains, with the exception of the addition of 0.2 mg L^−1^ in the strain UCD522 (P < 0.05). Similarly, the addition of fenbuconazole increased RS concentration in all fermentations with the exception of the addition of 0.2 mg L^−1^ in the strain EC1118 (P < 0.05). Indeed, RS concentrations were 34‐ to 185‐fold higher on average in all fermentations containing added fenbuconazole (Tables 1 and 2). Fermentations supplemented with fenbuconazole at a concentration of 0.4 mg L^−1^ averaged 8.6 g L^−1^ RS. This concentration is too high for the finished cider to be considered ‘dry’; therefore, these fermentations are considered incomplete or ‘stuck’, which is a term commonly used to describe incomplete fermentation in wine production. Even with cider styles where residual sugar is acceptable, stuck fermentation is not desirable because the outcome is unpredictable. Stopping the fermentation by chilling to stop yeast metabolism or centrifuging to remove yeast provides a more reliable means of producing cider that contains residual sugar. The addition of fenbuconazole at increasing concentrations shows linear correlations with fermentation rate, fermentation duration, CO_2_ evolution and residual sugar (Fig. 3). Increasing the fenbuconazole concentration resulted in a decreased fermentation rate, increased fermentation duration, decreased total CO_2_ evolved and increased residual sugar (P < 0.001). As such, the results of the present study indicate that residual fenbuconazole at 0.2 and 0.4 mg L^−1^ can adversely affect cider fermentation and the resulting cider quality.

The mechanism by which fenbuconazole impacts yeast growth and metabolism was not determined in the present study. However, the decreasing fermentation rate with an increasing concentration of fenbuconazole suggests that fenbuconazole may either lower cell viability, yeast cell biomass or yeast growth rate. The fungicide mode of action of fenbuconazole is through the inhibition of ergosterol production.^31^ Ergosterol is an essential cell membrane constituent in fungi and, if the enzymes that generate ergosterol are disrupted, cellular death may result.^32^ In practice, residual fenbuconazole in apple juice merits consideration as a possible cause of sluggish cider fermentation.

### Total H_2_S production

#### Elemental sulfur

**Figure 4:**
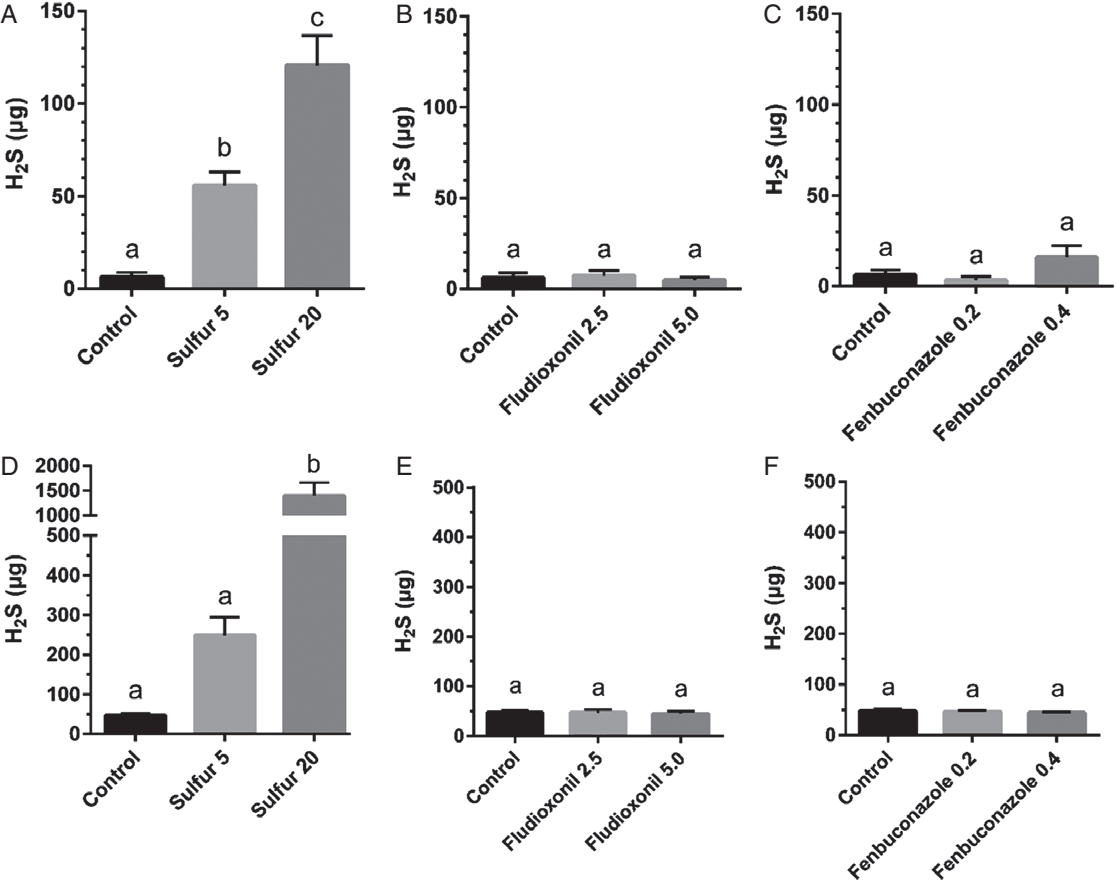
Total H_2_S produced during fermentations comparing treatments with fungicides at increasing concentrations. Values are expressed as the mean ± SD. Concentrations are expressed as µg H_2_S. Control fermentations contain no added fungicides. (A), (B) and (C) Fermentations using yeast strain EC1118. (D), (E) and (F) Fermentations using yeast strain UCD522. The y‐axis scales are not consistent because they reflect the maximum range of H_2_S produced within each yeast strain. Means not labeled with the same lowercase letter within specified yeast strain are significantly different (one‐way analysis of variance with Tukey's honestly significant difference, P < 0.05).

The addition of S^0^ to juice led to an increase in H_2_S production (Fig. 4). For yeast strain EC1118, H_2_S production increased nine‐fold on average when added at 5 mg L^−1^ S^0^ and 20‐fold on average when added at 20 mg L^−1^ S^0^. Similarly, for UCD 522, H_2_S production increased almost five‐fold on average when added at 5 mg L^−1^ and increased almost 30‐fold on average when added at 20 mg L^−1^. These results are in agreement with previous findings that have identified residual S^0^ as a significant contributor to H_2_S production.^15^, ^16^, ^17^ In white wine production, the practice of settling the juice prior to beginning fermentation has been shown to minimize the negative impacts associated with high concentrations of S^0^ in the fermenting medium.^33^ Pre‐fermentation juice settling is not a standard practice in cider production and cidermakers could potentially encounter higher residual S^0^ in juice that can increase H_2_S production. The concentration of residual S^0^ present on apples prior to processing and fermentations has not been assessed in the present study or by previous research. It is possible that orchards using S^0^ as a fungicide may encounter higher residual concentrations of S^0^ in juice compared to the residual concentrations found in grape juice or must prior to fermentation. All of these factors indicate that assessment of residual S^0^ on apples and the implementation of practices to prevent or limit residual S^0^ concentrations in apple juice pre‐fermentation is important in cider production.

#### Fludioxonil

The addition of fludioxonil did not result in increased H_2_S production for either yeast strain (Fig. 4). This finding is complementary to the findings on fermentation rate and duration, indicating that residual fludioxonil does not adversely affect yeast growth or metabolism in cider fermentation.

#### Fenbuconazole

The addition of fenbuconazole did not lead to increased H_2_S production (Fig. 4), although fenbuconazole did significantly impact fermentation kinetics (Fig. 2 and Tables 1 and 2). Yeast produce H_2_S through the sulfur reduction sequence (SRS).^34^ In this process, sulfate ions are converted to free sulfur (S^2−^), which is then used in yeast metabolism to produce sulfur‐containing amino acids such as methionine and cysteine.^34^ It is likely that fenbuconazole, at the concentrations tested in the present study, did not interfere with SRS signal transduction, and hence did not affect H_2_S production by yeast, even if it did impact cell growth and/or viability.

### Relative rate of H_2_S production

**Figure 5:**
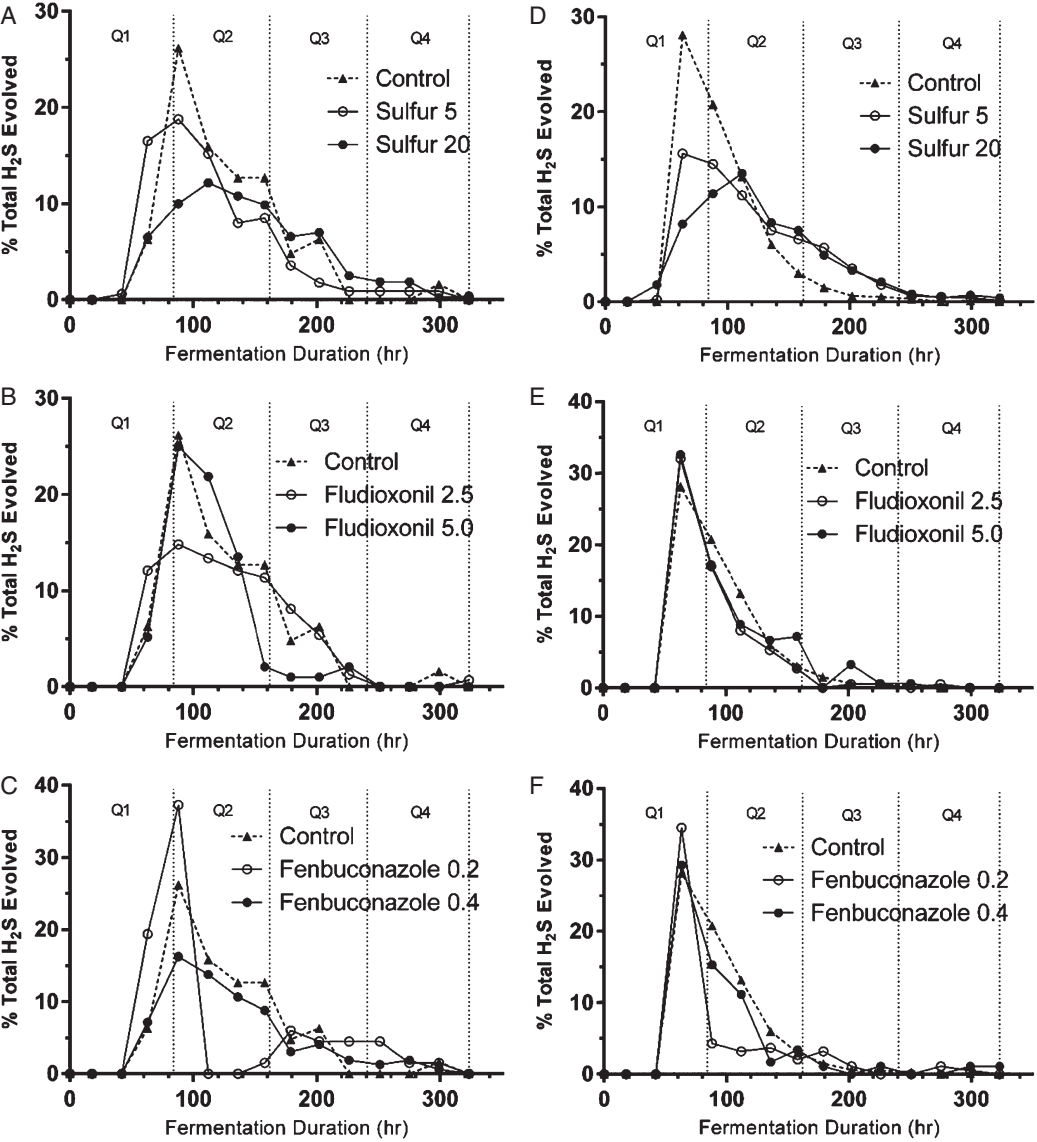
The percentage of the total H_2_S produced over the time course of fermentation. (A), (B) and (C) Ciders fermented using yeast strain EC1118. (D), (E) and (F) Ciders fermented using yeast strain UCD522. Q1–Q4 represent quartiles of the fermentation duration used to compare the rate of H_2_S evolution over the time course of fermentation.

The time course of H_2_S production during fermentation may vary because H_2_S can be effectively stripped from the fermenting media by CO_2_ gas evolved during fermentation,^35^ especially during the period of maximum fermentation rate when CO_2_ evolution is also at its maximum rate. It is possible that a cider in which less total H_2_S had been produced but was subsequently produced later in the time course of fermentation could retain more of the unwanted H_2_S character in the final product. This could impart a greater sensory impact on the finished product. The addition of S^0^, especially at high concentrations, tended to alter the rate of H_2_S production (Fig. 5A, D). The percentage of the total production of H_2_S for a given treatment was greater in the third and fourth quartiles at all concentrations of S^0^ in the strain UCD522 (*P* < 0.05) compared to the control. At concentrations of 20 mg L^−1^, H_2_S production was greater in the third quartile in the strain EC1118 (*P* < 0.01). The greatest proportion of the total H_2_S was produced in the second quartile for the majority of fermentations. This correlates with the logarithmic phase of fermentation, which also corresponds to the highest rate of SRS activity in the yeast, leading to the observable spike in H_2_S production during the highest rate of fermentation.^36^ When S^0^ is present in the fermenting juice, however, H_2_S is produced throughout the duration of the fermentation because SRS activity is not the primary cause of H_2_S production.^37^ The addition of fludioxonil did not affect the rate of H_2_S production (Fig. 5B, E). H_2_S production decreased in the second quartile when fenbuconazole was added at 0.2 mg L^−1^ in both yeast strains (*P* < 0.05) (Figs 4C, F). However, higher concentrations of fenbuconazole did not significantly impact total H_2_S production. It is possible that yeast cell density is correlated with a change in the relative rate of H_2_S production and that fenbuconazole may decrease total cell biomass achieved during fermentation. The mechanism by which fenbuconazole affects H_2_S production may be determined in future studies by monitoring cell density throughout the time course of fermentation and corresponding H_2_S production.

### Interactive effect of fenbuconazole and YAN

The fermentation kinetics results of the present study clearly illustrate that residual fenbuconazole can lead to sluggish and stuck cider fermentations. Insufficient YAN is also widely known to result in stuck and sluggish fermentations.^9^ The generally accepted minimum recommended concentration of YAN to successfully complete fermentation is 140 mg L^−1^ for wine.^38^ However, this recommendation is not universally accepted. The optimal concentration of pre‐fermentation YAN required for a given fermentation is difficult to determine as a result of interactions with numerous factors including but not limited to yeast strain,^1^, ^2^ micronutrient concentrations,^6^ biotin concentration,^6^ pantothenic acid concentration^6^ and temperature.^6^ The apple juice used in the present study contained an initial YAN concentration of 53 mg L^−1^ and was supplemented to 78 mg L^−1^ in all of the treatments. This relatively low YAN concentration is typical of apple juice.^39^ However, this concentration may still be insufficient to prevent sluggish or stuck fermentations and may have influenced the initial findings of the present study with respect to the impact of fenbuconazole on fermentation rate and duration.

**Figure 6:**
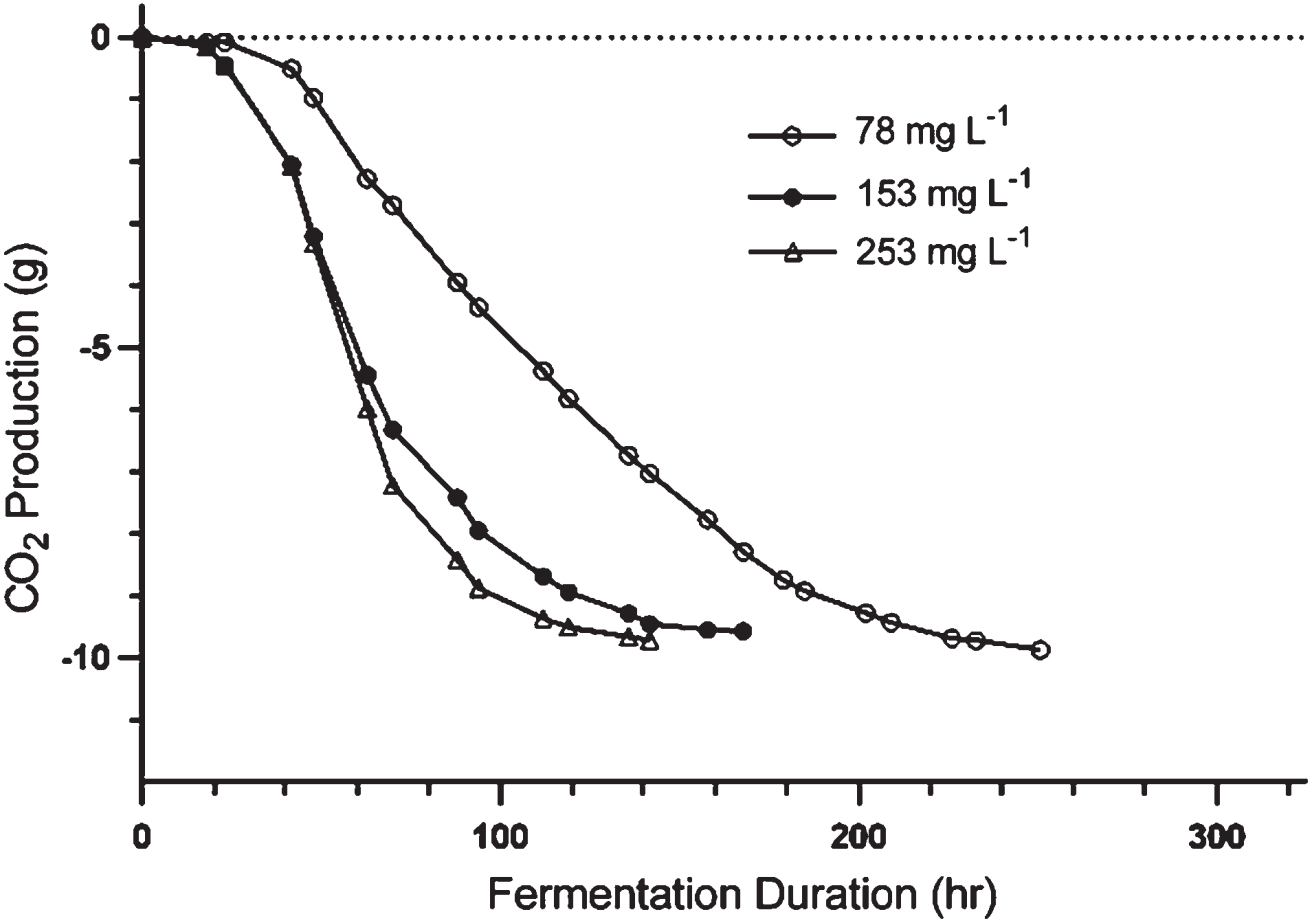
Fermentation curve for yeast strain EC1118 in apple juice containing different initial concentrations of YAN plotted as the mass of CO_2_ purged from the fermenter over the time course of fermentation. The slope of the curve during the logarithmic phase of growth was used to calculate and compare maximum fermentation rates. The fermentation rate increased significantly and fermentation duration decreased significantly with an increasing YAN concentration (one‐way analysis of variance with Tukey's honestly significant difference, P < 0.05).

**Figure 7:**
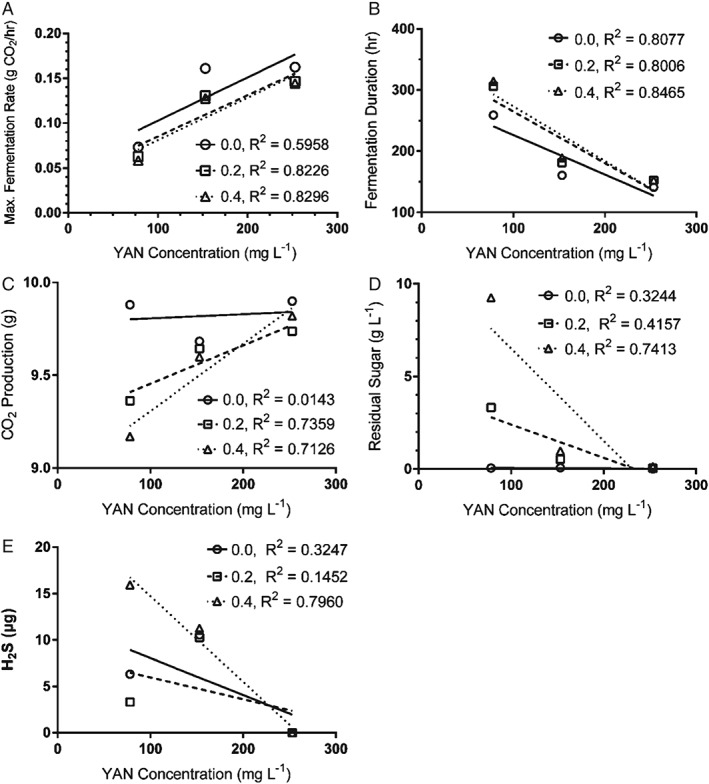
Linear regression of fermentation rate, fermentation duration, CO_2_ evolved, residual sugar and H_2_S production in fermentations treated with fenbuconazole and different concentrations of YAN. Concentrations of fenbuconazole are expressed as mg L^−1^. Linearity is significant for the fermentation rate at all concentrations (P < 0.05), fermentation duration at all concentrations (P < 0.01), CO_2_ evolution for concentrations of 0.2 and 0.4 mg L^−1^ (P < 0.01), residual sugar for concentrations of 0.4 mg L^−1^ (P < 0.01) and H_2_S production for concentrations of 0.4 mg L^−1^ (P < 0.01). There was a significant interaction between the addition of fenbuconazole and YAN for residual sugar (P = 0.0006) and H_2_S production (P = 0.0109).

The interactive effect of YAN and fenbuconazole on fermentations kinetics was investigated in fermentations conducted using yeast strain EC1118. One yeast strain and fungicide combination was selected to carry forward, with the experimental treatment now comprising an added residual fungicide concentration, YAN concentration, and the interaction of those two factors. The fermentation rate increased and fermentation duration decreased with an increasing YAN concentration (*P* < 0.05) (Fig. 6). This is in agreement with prior findings indicating that increasing YAN is correlated with increasing fermentation rate and decreasing fermentation duration.^8^ RS decreases with increasing YAN when fenbuconazole was present in concentrations of 0.4 mg L^−1^ (Fig. 7D), although RS was not significantly affected by increasing YAN for the control (no fenbuconazole) or at 0.2 mg L^−1^ fenbuconazole. This corroborates previous research indicating that increasing YAN minimizes sluggish or stuck fermentations,^8^, ^9^, ^10^ although the findings of the present study emphasize the influence of interactive effect of YAN with other parameters.

**Figure 8:**
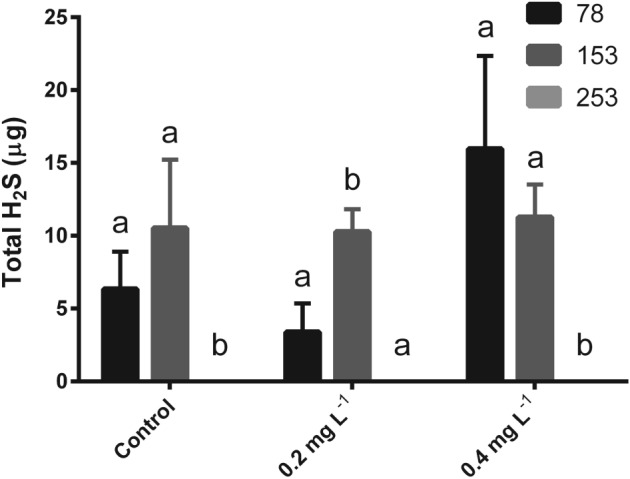
Total H_2_S produced during fermentations using yeast strain EC1118 comparing the interactive effects of total YAN and concentration of added fenbuconazole. Columns are grouped corresponding to the fenbuconazole concentration expressed as mg L^−1^. Values are expressed as the mean ± SD. Control juice contained no added fenbuconazole. Means not labeled with the same lowercase letter are significantly different within the specified fenbuconazole concentration (two‐way analysis of variance with Tukey's honestly significant difference, P < 0.05).

There was an interactive effect between the addition of fenbuconazole and YAN on H_2_S production (*P* < 0.0001) (Fig. 7E). Increasing the YAN from 153 to 253 mg L^−1^ decreased H_2_S production in all fermentations (*P* < 0.001) (Fig. 8). There was no detectable H_2_S when the YAN concentration was 253 mg L^−1^. H_2_S production decreases with an increasing YAN concentration at 0.4 mg L^−1^ fenbuconazole (Fig. 8), with both factors significantly correlating (*P* = 0.0012) (Fig. 7E). This corroborates previous findings indicating that insufficient YAN supplementation can lead to increased H_2_S production.^2^ The results of the present study show that a minimum YAN concentration of 140 mg L^−1^ does not always prevent the occurrence of stuck or sluggish fermentations. Furthermore, a minimum YAN concentration of 140 mg L^−1^ does not always prevent the formation of H_2_S during fermentation, even with the relatively low starting sugar concentration of apple juice (compared to grape juice, comprising the media in which most YAN investigations have been conducted).

The addition of both 0.2 and 0.4 mg L^−1^ fenbuconazole lowered the fermentation rate in juice at both 78 and 153 mg L^−1^ YAN (*P* < 0.05) (Table 3). However, there was no difference in fermentation rate for any concentration of fenbuconazole at 253 mg L^−1^ YAN. At YAN concentrations of 153 mg L^−1^, fermentations with added fenbuconazole at both concentrations demonstrated a slower fermentation rate than the control with no fenbuconazole (*P* < 0.01). However, fermentation duration was not different among fenbuconazole concentrations when the YAN concentration was 253 mg L^−1^. The interaction of YAN and fenbuconazole concentration was not significant in terms of fermentation rate or duration (Fig. 7A, B). With all other variables held constant, increasing YAN concentrations correlate with an increase in yeast biomass.^40^ It is possible that residual fenbuconazole inhibited yeast growth and promoted yeast mortality. One possible explanation for the results of the present study is that increasing the YAN concentration may overcome this effect by allowing yeast biomass to increase to the point where the impact of residual fenbuconazole on yeast cell populations becomes negligible. Interestingly, fenbuconazole still adversely impacted fermentation kinetics when the YAN concentration was 153 mg L^−1^, which is higher the minimum concentration 140 mg L^−1^ typically considered sufficient to complete fermentations in wine and cider. Therefore, the minimum YAN concentration required for fermentation is greater than the standard recommendation when fenbuconazole is present in the juice.

**Table 3:** Fermentation rate, fermentation duration, total CO_2_ production and residual sugar for interactive fermentations of fenbuconazole and YAN

YAN	Fenbuconazole concentration	Fermentation rate (g CO_2_ h^−1^)	Fermentation duration (h)	Total CO_2_ production (g)	Residual sugar (g L^−1^)
	0.0	0.071 ± 0.001 a	259 ± 0 a	9.90 ± 0.09 a	0.07 ± 0.01 a
78	0.2	0.065 ± 0.001 b	318 ± 9 b	9.94 ± 0.13 a	2.40 ± 1.19 b
	0.4	0.061 ± 0.001 c	322 ± 13 b	9.41 ± 0.06 b	7.72 ± 0.99 c
	0.0	0.141 ± 0.000 a	160 ± 4 a	9.69 ± 0.12 a	0.07 ± 0.03 a
153	0.2	0.131 ± 0.003 ab	181 ± 0 b	9.64 ± 0.10 a	0.53 ± 0.15 b
	0.4	0.128 ± 0.003 b	189 ± 0 b	9.63 ± 0.13 a	0.97 ± 0.27 c
	0.0	0.163 ± 0.001 a	141 ± 0 a	9.90 ± 0.12 a	0.01 ± 0.00 a
253	0.2	0.146 ± 0.004 a	152 ± 10 a	9.74 ± 0.09 a	0.05 ± 0.02 a
	0.4	0.144 ± 0.005 a	152 ± 10 a	9.82 ± 0.05 a	0.13 ± 0.01 a

Values expressed as the mean ± SD.

Values marked with different lowercase letters are significantly different from the control within a specified fungicide treatment (ANOVA with Tukey's HSD).

Fungicide concentrations are expressed as mg L^−1^.

The RS concentration increased with increasing concentrations of fenbuconazole at YAN concentrations of 78 mg L^−1^ and 153 mg L^−1^ (Table 3). However, with a concentration of 153 mg L^−1^ YAN, the RS concentration was ten‐ to 20‐fold lower compared to juices with the same fenbuconazole concentration fermented at 78 mg L^−1^ YAN. When the YAN concentration was 153 mg L^−1^, all treatments fermented completely. Finally, there was no difference in RS concentration at any concentration of fenbuconazole when the YAN concentration was 253 mg L^−1^ (Table 3). There was an interaction effect of YAN and fenbuconazole on RS (*P* = 0.0006) (Fig. 7D).The effect of fenbuconazole on RS at a low YAN concentration becomes negligible at high YAN concentrations (Fig. 7D). This further corroborates the finding that increasing YAN lessens the impact of fenbuconazole on yeast growth and metabolism. There was no difference in CO_2_ when fenbuconazole was added to juice with153 or 253 mg L^−1^ YAN (Table 3). Even though CO_2_ production did not decrease with an increasing YAN from 153 to 253 mg L^−1^, there was a significant correlation between an increasing YAN and a decreasing CO_2_ when fenbuconazole was added at either concentration (*P* < 0.01) (Fig. 7C). This supports the hypothesis of the present study suggesting that increasing the YAN concentration contributes to an increased fermentation rate, even when fenbuconazole is present. The addition of fenbuconazole did not affect H_2_S production for any of the YAN treatments.

## CONCLUSIONS

As a result of the complex chemical nature of the apple juice/cider matrix and the interactive effects of many factors on fermentation performance, it is difficult to determine the complete impact of fungicide residues on cider fermentation and product quality. Notwithstanding, the present study showed that fungicide residues can impact fermentation rate, particularly under conditions of low YAN concentration. Specifically, residues of fenbuconazole should be of specific concern to cidermakers, even at low concentrations. Fludioxonil applications for the post‐harvest storage of apples are not likely to negatively affect fermentation kinetics during cider fermentation. Fermentation kinetics and H_2_S production within a given yeast strain differed across fungicides in the present study, likely as a result of differences in fungicide chemistry and the mode of action. Because fungicide applications differ widely across fruit production systems and regions, this observation is of special concern to emerging apple and grape growing regions that contend with relatively high fungal disease pressure, such as the Eastern USA. The results of the present study indicate that the presence of fungicide residues should be added to the growing list of factors beyond starting Brix, which should be taken into consideration when determining the minimum YAN concentration for cider fermentation. Determining regionally specific guidelines for minimum YAN concentration in cider will require further research into the effects on fermentation rate and H_2_S production of interactions between YAN and fungicide residues, as well as other juice matrix factors.
